# Recurrent Coronary Thrombus in a Patient with Chronic Immune Thrombocytopenia with Treatment Using Eltrombopag

**DOI:** 10.1155/2019/2756319

**Published:** 2019-03-26

**Authors:** Terumori Satoh, Masao Saotome, Kenichiro Suwa, Hayato Ohtani, Yasuyuki Nagata, Takaaki Ono, Yuichiro Maekawa

**Affiliations:** ^1^Department of Cardiology, Internal Medicine 3, Hamamatsu University School of Medicine, Hamamatsu, Japan; ^2^Department of Hematology, Internal Medicine 3, Hamamatsu University School of Medicine, Hamamatsu, Japan

## Abstract

**Background:**

Eltrombopag, a nonpeptide thrombopoietin receptor agonist (TPO-RA), has been reported to be an effective therapy for chronic immune thrombocytopenia (ITP). However, a higher incidence of arterial and venous thromboembolic events was reported after using eltrombopag.

**Case Presentation:**

A 67-year-old man, treated with eltrombopag due to chronic ITP, was admitted due to acute coronary syndrome (ACS). Although coronary angiography revealed no occlusion, cardiac magnetic resonance imaging suggested a myocardial infarction in the territory of the left circumflex coronary artery. Three months after the ACS event, the obtuse marginal branches exhibited significant stenosis; hence, a percutaneous coronary intervention (PCI) was performed to implant a zotarolimus-eluting stent under the treatment of a dual antiplatelet therapy. However, stent thrombosis occurred 3 hours after PCI and required three other PCIs during the eltrombopag treatment.

**Conclusion:**

We present a case of an ITP patient, who experienced repeated coronary and stent thrombosis during the treatment with eltrombopag. We propose that the risk of ACS and consequent coronary stent thrombosis should be considered before the introduction of eltrombopag.

## 1. Introduction

Chronic immune thrombocytopenia (ITP) is an autoimmune syndrome, which is caused by the development of an autoantibody against structural platelets. Although bleeding is a major symptom of ITP, a thrombophilia condition can be promoted in ITP patients by excess activated platelet function. Since platelets play critical roles in the progression of atherosclerosis, cardiovascular events have often been reported in ITP patients. Thus, the safety and recommended strategy in percutaneous coronary intervention (PCI) and subsequent antiplatelet therapies toward ITP patients have remained a matter of debate.

Currently available treatments of ITP include corticosteroids, splenectomy, immunosuppressants, and thrombopoietin receptor agonist (TPO-RA). Eltrombopag, a nonpeptide TPO-RA, has been reported to be effective as a second-line therapy for ITP. However, previous reports indicated a higher incidence of arterial and venous thromboembolic events, including myocardial infarction, after using eltrombopag [1]. In this report, we showed a case of recurrent coronary thrombosis in a patient with ITP who was treated with eltrombopag. We also reviewed the previous reports of PCI and antiplatelet therapy, during and after PCI, in ITP patients.

## 2. Case Presentation

**Figure 1:**
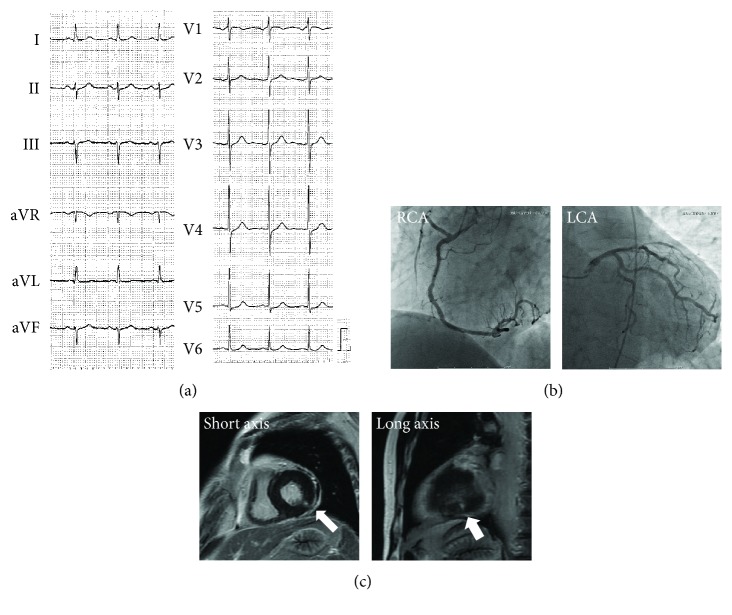
Electrocardiography (ECG), coronary angiography (CAG), and cardiac magnetic resonance imaging (C-MRI) on the first admission. (a) ECG on admission revealed no significant change except a left axis deviation. (b) CAG revealed no coronary artery occlusion either in the right coronary artery (RCA: left anterior oblique 50° view) or the left coronary arteries (LCA: caudal 30° view). (c) C-MRI of short and long axis view. The arrows indicate the delayed enhancement of the myocardium.

A 67-year-old man, who was suffering from long-lasting (>2 hours) severe chest pain, was admitted in our hospital. He was an ex-smoker (between the ages of 20 and 25 years) and had been treated for hypertension and hyperlipidemia. He was diagnosed with ITP 6 months before admission and maintained his platelet count between 10.0 × 10^9^/L and 15.0 × 10^9^/L by receiving prednisolone (10 mg daily) and eltrombopag (50 mg daily). He was immediately relieved of chest pain after admission, and there was no significant finding on a 12-lead electrocardiogram except a left axis deviation (Figure 1(a)). However, a cardiac catheterization was performed urgently due to the significant increase of serum myocardial enzymes (troponin-I 1.56 ng/mL, creatine kinase (CK) 233 IU/L, and CK-muscle/brain 20 IU/L). Although coronary angiography (CAG) revealed no artery occlusion (Figure 1(b)), left ventriculography showed mild hypokinesis in the posterior wall, and cardiac magnetic resonance imaging exhibited a delayed gadolinium enhancement in the posterior wall area (Figure 1(c)), suggesting myocardial infarction in the territory of the left circumflex coronary artery (LCX).

**Figure 2:**
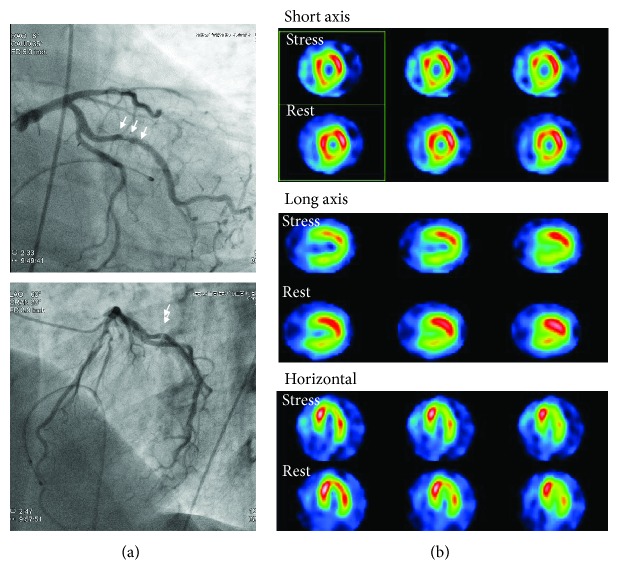
Second CAG and adenosine stress myocardial perfusion scintigraphy. (a) The second CAG was performed to investigate the acetylcholine-induced vasospasm. Although the coronary arteries did not exhibit vasospasm induced by acetylcholine, CAG revealed de novo stenosis in #12. (b) The adenosine stress myocardial perfusion scintigraphy (Tc-99m tetrofosmin) showed the myocardial ischemia in the posterior wall area, suggesting myocardial ischemia in #12.

**Figure 3:**
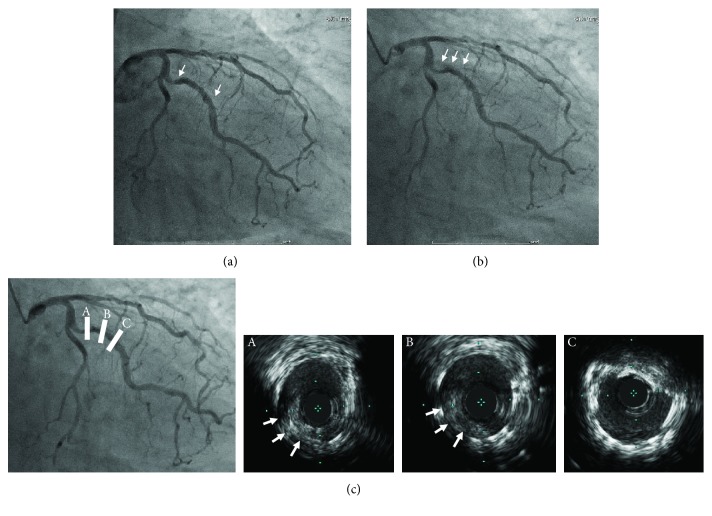
CAG and intravascular ultrasound images during PCI. In the first PCI, a zotarolimus-eluting stent (3.0 × 12 mm) was implanted in #12 (a, between white arrows). Emergent CAG, which was performed 3 hours after the first PCI, revealed massive thrombus in the stent lesion (b, white arrows). The intravascular ultrasound study (c) showed the new lining thrombus (white arrows) in the stent proximal (A and B), whereas not in the distal (C).

**Figure 4:**
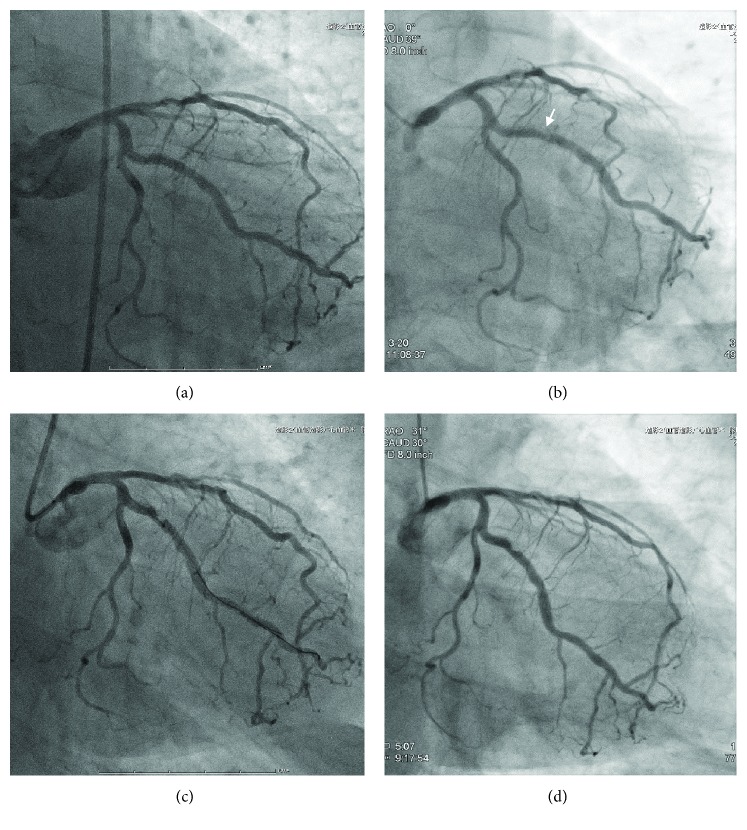
CAG findings after second PCI. Although an additional drug-eluting stent sized 3.0 × 12 mm was implanted in #12 (a), CAG performed 3 days after the second PCI revealed the recurrence of thrombus (white arrow) with 75% haziness stenosis in #12 (b). Another CAG performed 14 days after the third PCI again revealed recurrence of stent thrombosis with 75% stenosis in #12 (c). The final CAG, which was performed after the fourth PCI, showed 50% of residual stenosis with thrombus, and we decided not to perform further PCI to #12 (d).

Because the patient persistently complained of chest pain even after discharge, the second CAG was performed 3 months after the discharge. CAG revealed de novo stenosis in the obtuse marginal branches (#12: 75% with haziness, Figure 2(a)), and adenosine stress myocardial perfusion scintigraphy (Tc-99m tetrofosmin) showed myocardial ischemia in the posterior wall area (Figure 2(b)), where the tracer uptake was reduced at stress and restored at rest. Thus, the first PCI was planned for #12, and a dual antiplatelet therapy (aspirin 100 mg daily and clopidogrel 75 mg daily) was started more than 1 month before the procedure, where his platelet was kept at 27.4 × 10^9^/L by eltrombopag (50 mg daily). The first PCI toward #12 was successfully performed by implantation of a zotarolimus-eluting stent (3.0 × 12 mm) and CAG revealed no residual stenosis (Figure 3(a)). However, the patient complained of severe chest pain with significant ST depression after returning to his hospital room (3 hours after the PCI). The emergent CAG demonstrated a stent thrombosis at the proximal edge of the stent (Figure 3(b)). Although the intravascular ultrasound imaging exhibited neither the underexpansion of the prior-implanted stent nor the coronary artery dissection around the stent, it showed new lining thrombus (white arrows) at the proximal edge of the stent (Figure 3(c)). An additional drug-eluting stent (3.0 × 12 mm, everolimus-eluting stent) was implanted at the proximal edge of the prior stent lesion of #12 (Figure 4(a), second PCI) under the support of an intra-aortic balloon pumping. To avoid further stent thrombosis, the antiplatelet agent was changed from clopidogrel (75 mg daily) to prasugrel (3.75 mg daily). However, CAG, performed 3 days after the second PCI, revealed a massive thrombus with 75% stenosis in #12 (Figure 4(b)). Urokinase (UK 240.000 U) was selectively injected into the LCX, instead of balloon inflation for the third PCI, because recurrent coronary thrombosis was occurring at the area of the dual stent implantation. CAG after the UK injection revealed the residual stenosis (50%) in #12. The follow-up CAG, which was performed 14 days after the third PCI, again revealed the recurrence of stent thrombosis with 75% stenosis in #12 (Figure 4(c)). Subsequently, a coronary thrombus aspiration was performed as the fourth PCI. The final CAG after coronary thrombus aspiration exhibited 50% residual stenosis. Since his platelet maintained at >39.3 × 10^9^/L by eltrombopag during all PCIs, the association between a recurrence of coronary thrombosis and inappropriate platelet increase induced by eltrombopag was suspected. Thus, we transiently ceased eltrombopag, and his platelet promptly decreased to 8.0 × 10^9^/L after 7 days of cessations. CAG, after the cessation of eltrombopag, showed 50% of residual stenosis with thrombus; hence, we did not perform further PCI to #12 (Figure 4(d)). In addition, after he received a splenectomy to cease eltrombopag, he has not complained of any chest pain, and CAG after splenectomy revealed no thrombus in #12.

## 3. Discussion and Conclusions

We report a rare case of recurrent coronary thrombosis in a patient with ITP who was treated with eltrombopag. Despite the thrombocytopenic condition, acute coronary syndrome (ACS) events have been reported in ITP patients. ITP patients could experience comorbid thromboembolic events (TEEs) because ITP itself affords an inflammatory condition and promotes an aberrant platelet condition, with larger, younger, and more adherent platelets. In addition, the multifactorial predisposing factors, such as prednisolone treatment and atherosclerotic comorbidities, facilitate the trigger of TEEs.

Although eltrombopag has been commonly used as a second-line therapy of ITP, it may enhance the ITP-specific characteristics of thromboembolism, which may increase the incidence of ACS events in ITP patients. Previous reports described cardiac manifestation, such as tachycardia, cyanosis, QT interval prolongation, pulmonary thromboembolism, and ACS after the use of eltrombopag. A pooled data analysis from five eltrombopag RCTs exhibited that the overall TEE rate was 4.5% (27 cases) in the eltrombopag-treated ITP patients, whereas there was none in the placebo groups, and four patients had ACS event [2]. Previously, three ACS events in patients with ITP were reported, which are suspected to be associated with eltrombopag. No association seems to be seen in the ACS event with the treatment duration (1 to 12 months), the platelet counts (107 to 437 × 10^9^/L), and the atherosclerotic risk factors among them [3–5]. Although the mechanism, by which eltrombopag causes ACS event, remains elusive, a rapid platelet increase induced by eltrombopag (0.8 × 10^9^/L to 10.5 × 10^9^/L per month) may affect the ACS events in the present case.

Since platelets play critical roles in the progression of atherosclerosis, as well as vascular healing, after stent implantation, PCI and the antiplatelet therapies in ITP patient have been a matter of debate for a long time [6]. The present case showed recurrent coronary thrombosis in the LCX even under dual antiplatelet therapy (DAPT—both clopidogrel plus aspirin and prasugrel plus aspirin), suggesting that DAPT may not be effective for activated platelets induced by eltrombopag. Thus, when ITP patients using eltrombopag have a coronary event, it might be safe to avoid any nonurgent PCI. If possible, PCI should be performed with other supporting treatments, such as corticosteroids, splenectomy, and immunosuppressants. Even in an evitable condition, such as ACS, it might be better to avoid any implantation of drug-eluting stent because they might have a poor response to DAPT, which results in repetitive coronary thrombosis. Furthermore, even in ITP patients not receiving eltrombopag treatment, adding eltrombopag before PCI is not recommended. The DAPT treatment for ITP patients has been commonly accepted if ITP patients have a sufficient platelet count (>50 × 10^9^/L) [7]. Even in severe thrombocytopenic conditions, major bleeding was rarely reported in the previous cases [6].

Accordingly, when ITP patients present any risk factors for thromboembolism or atherosclerosis, we recommend careful consideration of the risk-benefit balance before the introduction of eltrombopag. Further investigation is required to detect the eltrombopag-specific factors which increase the TEEs.

## Abbreviations

ACS: Acute coronary syndrome
CAG: Coronary angiography
CK: Creatine kinase
DAPT: Dual antiplatelet therapy
ITP: Immune thrombocytopenia
LCX: Left circumflex coronary artery
TPO-RA: Thrombopoietin receptor agonist
TEEs: Thromboembolic events.
